# A single amino acid substitution in CXCL12 confers functional selectivity at the beta-arrestin level

**DOI:** 10.18632/oncotarget.25533

**Published:** 2018-06-22

**Authors:** Antonella Rigo, Isacco Ferrarini, Giulio Innamorati, Fabrizio Vinante

**Affiliations:** ^1^ Section of Hematology, Cancer Research & Cell Biology Laboratory, Department of Medicine, University of Verona, Verona, Italy; ^2^ Laboratory of Translational Surgery, Department of Surgical Sciences, Dentistry, Gynecology and Pediatrics, University of Verona, Verona, Italy

**Keywords:** CXCL12, [N33A]CXCL12, G protein-coupled receptors, biased agonism, β-arrestin

## Abstract

CXCL12/CXCR4 axis relies on both heterotrimeric G_i_ protein and β-arrestin coupling to trigger downstream responses. G protein activation allows for calcium flux, chemotaxis and early extracellular-signal regulated kinases 1/2 (ERK1/2) phosphorylation, whereas β-arrestin recruitment leads to late signaling, receptor desensitization and internalization. Together they may regulate the balance between transactivation and transinhibition of epithelial growth factor receptor 1 (HER1). Since we have previously noted significant differences between CXCL12 and its structural variant [N33A]CXCL12 in CXCR4 signaling, we sought to better characterize them by performing cAMP inhibition and β-arrestin recruitment assays, as well as functional tests that separately investigate G protein and β-arrestin-induced responses. [N33A]CXCL12 showed reduced potency both in Gα_i_ coupling and β-arrestin recruitment as compared to the wild type chemokine, acting as an unbiased ligand. While these findings translated into reduced potency within Gα_i_-dependent functions, β-arrestin-dependent modules were affected in a more peculiar way. Unlike CXCL12, the mutant analogue did not restore HB-EGF-stimulated HER1 from CXCR4-induced transinhibition, and did not trigger the late wave of ERK1/2 phosphorylation. Instead, CXCR4 internalization was not impaired upon [N33A]CXCL12 stimulation. These differences highlight the novel opportunity to dissect CXCL12 signaling within the β-arrestin layer, in which the mutant chemokine clearly favors the internalization module over the other pathways. Such functional selectivity has an impact on HER1 activation status and may play a relevant part in the crosstalk between tyrosine kinase and seven transmembrane receptors.

## INTRODUCTION

CXCL12 is a CXC homeostatic chemokine, constitutively expressed by blood and stromal cells, which retains a central role in the regulation of embryogenesis [1], hematopoietic system [2], tissue repair mechanisms [3] and cancer biology [4]. It specifically interacts with the seven transmembrane receptors (7TMRs) CXCR4 and CXCR7 to generate a complex network of intracellular biochemical signals. While CXCR7, now renamed atypical chemokine receptor 3 (ACKR3), is considered a decoy receptor which efficiently mediates ligand internalization but fails to induce classical chemokine signaling [5], CXCR4 couples to G proteins and β-arrestin to elicit pivotal responses in the context of cell migration, proliferation and survival [6]. It is widely recognized that upon CXCL12 stimulation CXCR4 intracellular loop 3 (ICL3) changes its conformation to permit Gα_i_ activation and release of free βγ subunits, which in turn inhibit adenylyl cyclase, stimulate mitogen-activated protein kinase (MAPK) pathway and initiate calcium-dependent biochemical cascades [7]. In more recent years, α subunits other than α_i_ have been demonstrated to couple to CXCR4, such as α_q_ and α_12/13_, thus triggering phospholipase C (PLC) and Rho module activation, respectively, and contributing to the fine tuning of chemotaxis and homing [8, 9]. Apart from classical G protein signaling, CXCL12 induces G protein-coupled receptor kinases (GRK)-mediated phosphorylation of CXCR4 cytoplasmic tail and subsequent recruitment of β-arrestin proteins [10, 11]. They are traditionally involved in canonical signaling termination by sterically hindering G protein coupling to the receptor and in facilitating its internalization by acting as adaptors for the endocytosis-promoting molecules clathrin and β-adaptin. However, recruited β-arrestins can also function as scaffolds for multiprotein complexes, which initiate signal transduction by themselves. This mechanism accounts for the persistence of extracellular-signal regulated kinases 1/2 (ERK1/2) phosphorylation after the first, pertussin toxin-sensitive, spike, as well as the activation of AKT, PI3K and phosphodiesterase 4 (PDE4) [12–14].

Within the intricate complexity of G protein-dependent and arrestin-dependent signals, the concept of biased ligands, i.e. ligands exhibiting functional selectivity for one pathway versus another, has emerged as a fascinating way to dissect intracellular responses [15]. Since several endogenous biased ligands have been identified, it seems likely that functional selectivity has evolved for fine regulation in complex signaling systems. For instance, CCL19 and CCL21, two endogenous ligands for CCR7, exhibit similar G protein coupling efficacy but differ greatly in arrestin binding, receptor desensitization and ERK1/2 phosphorylation [16]. Instead, angiotensin-(1-7) acts as a natural β-arrestin biased agonist at the AT-1 receptor, thus preventing the signaling branch leading to ventricular hypertrophy [17].

CXCL12/CXCR4 axis plays a major role in the pathophysiology of solid and hematological cancers [4]. In cancer microenvironment, both neoplastic and surrounding immune cells may express functional CXCR4, so the downstream G protein and arrestin dependent signaling modules can exert pleiotropic tumor-promoting effects, such as favoring chemotaxis in a Rac1 dependent manner [9], recruiting endothelial progenitors to initiate neoangiogenesis [18] and shaping macrophage polarization towards the immune tolerant M2 phenotype [19]. Taking into account the multiple faces of CXCL12 biological effects in both neoplastic and physiological conditions, searching for CXCR4 biased ligands, which could preferentially activate a specific pathway, is becoming a matter of interest [20].

In a previous work, we have demonstrated that, as compared to the wild type chemokine, the chemically synthetized mutant [N33A]CXCL12 (N33A) was not able to reverse G protein-dependent transinhibition of the heparin-binding EGF-like growth factor (HB-EGF)-stimulated epithelial growth factor receptor 1 (HER1), amounting to an indirect proof that it might stabilize CXCR4 in a conformation favoring G protein signaling over β-arrestin recruitment [21]. However, we could not provide definitive evidence about N33A-induced selectivity mechanisms. In the present study, we tried to better characterize the differences between the mutant variant and the wild type chemokine in G protein coupling and β-arrestin binding, and we set out a series of experiments on Jurkat and HeLa cells, endogenously expressing CXCR4, to investigate G protein and arrestin-dependent functions. Surprisingly, we found that N33A is a CXCR4 partial agonist inducing no bias towards G protein coupling over β-arrestin recruitment. Instead, the substantial differences that we have pointed out between CXCL12 and N33A at the level of β-arrestin-induced responses strongly suggest a functional selectivity towards distinct arrestin-dependent modules, providing an opportunity to dissect CXCL12 signaling within the β-arrestin layer.

## RESULTS

### N33A shows reduced potency in coupling to Gα_i_

**Figure 1:**
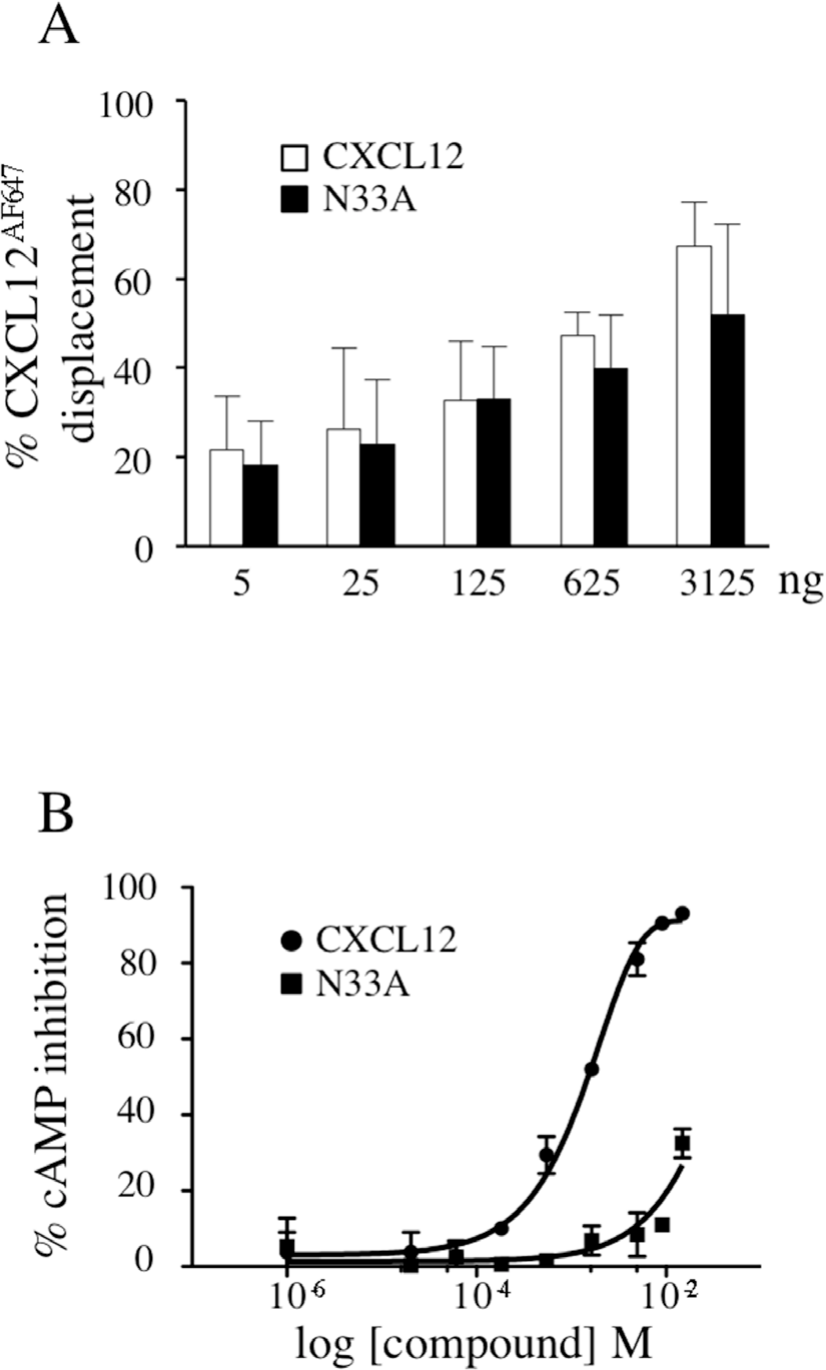
CXCL12 and N33A binding ability to CXCR4 and Gαi-coupling efficacy **(A)** Jurkat cells were incubated with CXCL12^AF647^, washed and the indicated concentrations of CXCL12 or N33A were added. The percentages of CXCL12^AF647^ displacement by the unlabeled chemokines were reported. **(B)** Activation of Gα_i_ by the CXCR4 was measured at 15 minutes in CHO HitHunter^®^ cells by assessing inhibition of forskolin-mediated cAMP production in the presence of increasing concentrations of CXCL12 or N33A. EC_50_ 16.4 nM and 1.4 nM, respectively. E_max_ 100% and 32%, respectively. Means ± SD of at least 3 experiments are depicted. p<0.05.

CXCR4 classically couples to Gi subfamily of heterotrimeric G proteins. Upon CXCL12 stimulation, CXCR4 signaling pathway begins with α_i_ and βγ subunits dissociation and subsequent Gα_ι_-mediated adenylyl cyclase inhibition [6]. We first wondered whether the chemical mutant N33A exhibited different potency and efficacy in coupling to Gα_i_ as compared to the wild type form. CXCL12 and N33A had the same binding affinity to CXCR4, as the percentage of displacement of the fluorescinated analogue CXCL12^AF647^ was the same for the two molecules (Figure 1A). By performing cAMP inhibition assay at 15 minutes in CXCR4-expressing CHO cell line, we observed that EC_50_ was higher, whereas E_max_ was reduced for N33A as compared to CXCL12, thus demonstrating that N33A acts as a weak partial agonist in coupling to Gα_i_ (Figure 1B).

### N33A exhibits reduced potency in G protein-dependent functions

**Figure 2:**
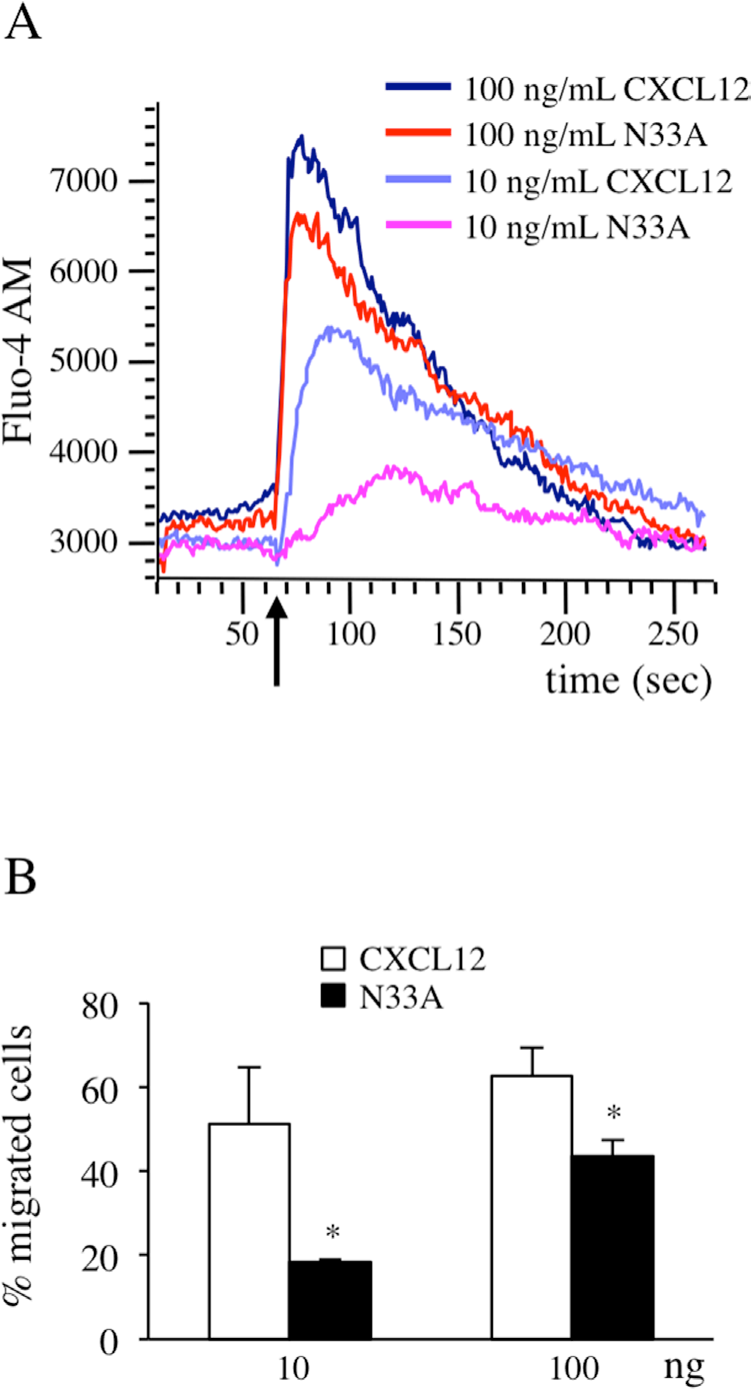
CXCL12 and N33A exhibit different potency in G protein-dependent functions in Jurkat cells Cells were stimulated by CXCL12 or N33A at the indicated concentrations. **(A)** Flow cytometry detection of cellular Ca^++^ concentration revealed by Fluo-4 AM probe. Arrow indicates the addition of the stimulus. A representative case is depicted out of four experiments. **(B)** Evaluation of cell chemotaxis at 90 minutes through 8-μM pore transwell filters. Means ± SD of at least 5 experiments are depicted. ^*^p<0.05.

After G protein coupling, the exchange of GTP for GDP on the α subunit induces conformational changes that lead to βγ complex dissociation and signaling initiation. Both Gα_i_ and Gβγ subunits promote phospholipases activation to trigger intracellular calcium flux [6], which can be regarded as a universal signal transducer involved in the regulation of a wide number of physiological processes, such as gene transcription, cell proliferation and migration [22, 23]. To investigate the differences in calcium flux induction between CXCL12 and its mutant variant, we loaded Jurkat cells, endogenously expressing CXCR4, with the green fluorescent calcium indicator Fluo-4 AM and recorded the results over a time period of 250 seconds. At low concentrations, N33A was less potent than CXCL12 in mobilizing intracellular calcium, whereas the differences attenuated when reaching concentrations closed to 100 ng/mL (Figure 2A).

Since chemotaxis is one of the best characterized G protein-dependent cell responses [6], resulting from tight regulation of integrin system activation and cytoskeletal rearrangements [24], we chose it as an indirect measure of G protein-dependent activation pathways. We performed chemotaxis assays by stimulating Jurkat cells with CXCL12 or N33A. In contrast with a previous work [25], the mutant form was less potent than the wild type one in inducing cell migration (Figure 2B).

### N33A shows reduced potency in recruiting β-arrestin

**Figure 3:**
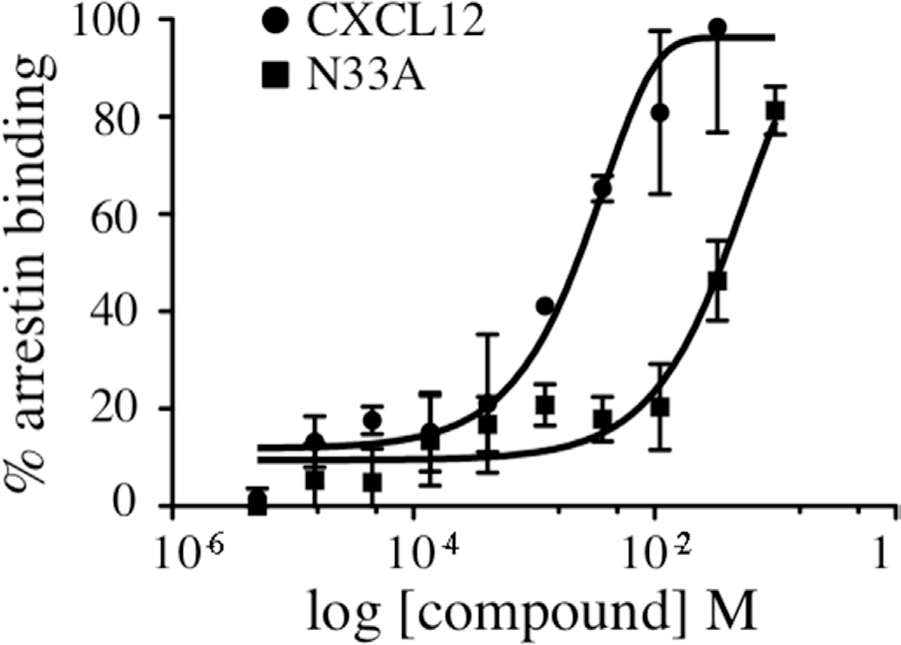
Recruitment of β-arrestin to CXCR4 Arrestin binding to CXCR4 was measured in CHO-PathHunter^®^ cells in the presence of increasing concentrations of CXCL12 or N33A at 15 minutes. EC_50_ 39.98 nM and 3.1 nM, respectively. Means ± SD of at least 3 experiments are depicted. ^*^p<0.05.

Upon agonist stimulation, 7TMRs change their multidimensional conformation and become substrates for GRK-mediated phosphorylation, which in turn promotes arrestin recruitment to the phosphorylated C-terminus tail [26]. Since ligand structure can affect intracellular conformational changes, we used the PathHunter β-arrestin assay to evaluate if there were some differences between CXCL12 and N33A in β-arrestin recruitment. As shown by dose-response curve at 15 minutes (Figure 3), the mutant chemokine had reduced potency in β-arrestin binding as compared to the wild type one.

To evaluate whether N33A exhibited functional selectivity towards β-arrestin recruitment or G protein engagement, τ and K_A_ values were deduced from dose-response curves fitted using the operational model-partial agonist function. τ (τ = [Rt]/K_E_) describes the ligand's efficacy towards specific pathways and is defined by the ratio of the total receptor density ([Rt]) over the general equilibrium dissociation constant (K_E_). K_A_ is defined as the ligand equilibrium dissociation constant of the ligand-receptor complex. By applying the method published by Kenakin [27], the transduction ratios (τ/K_A_) and the transduction coefficients [Log (τ/K_A_)] for CXCL12 and N33A were deduced for each pathway; we calculated the ΔLog(τ/K_A_), ΔLog(τ/K_A_) = Log(τ/K_A_)_N33A_ − Log(τ/K_A_)_CXCL12_, for each of the pathways considered (Gα_i_ engagement and β-arrestin recruitment to CXCR4). To quantify the N33A bias for β-arrestin recruitment *vs* Gα_i_ engagement, we calculated the ΔΔLog (τ/K_A_) as a difference between the ΔLog(τ/K_A_) values obtained for N33A for both signaling pathways, ΔΔLog(τ/K_A_) = ΔLog(τ/K_A_)CXCL12/N33A_β-arrestin_ − ΔLog(τ/K_A_)CXCL12/N33A_Gαi_. The bias factor, calculated as the anti-Log value of the ΔΔLog(τ/K_A_), was 1.04±0.27 (Table 1).

**Table 1:** Data describing the absence of significant bias of N33A for Gα_i_ coupling and β-arrestin recruitment to CXCR4

Engagement/Recruitment	τ	K_A_	Log(τ/K_A_)±SEM	ΔLog(τ/K_A_)±SEM	ΔΔLog(τ/K_A_)±SEM	Bias±SEM
**Gα_i_ coupling**						
CXCL12	59.30	54.68	0.04±0.01			
N33A	1.10	0.06	1.26±0.07	1.22±0.07		
**β-arrestin recruitment**						
CXCL12	4.00	3.59	0.05±0.19		0	
N33A	1.17	0.06	1.29±0.03	1.24±0.16	0.02±0.23	1.04±0.27

Collectively, these results show that N33A is a weaker CXCR4 agonist, but fail to bring out any evidence in favor of functional selectivity towards either G protein or β-arrestin pathway.

### CXCL12 and N33A activate distinct β-arrestin dependent modules

When β-arrestin binding to the receptor cytoplasmic tail occurs, at least three different arrestin-dependent events are triggered: termination of G protein-derived responses, initiation of G protein-independent signaling and clathrin-mediated receptor endocytosis [28]. Though globally less potent in β-arrestin recruitment, we sought to investigate whether N33A could preferentially elicit one arrestin-dependent module instead of another.

**Figure 4:**
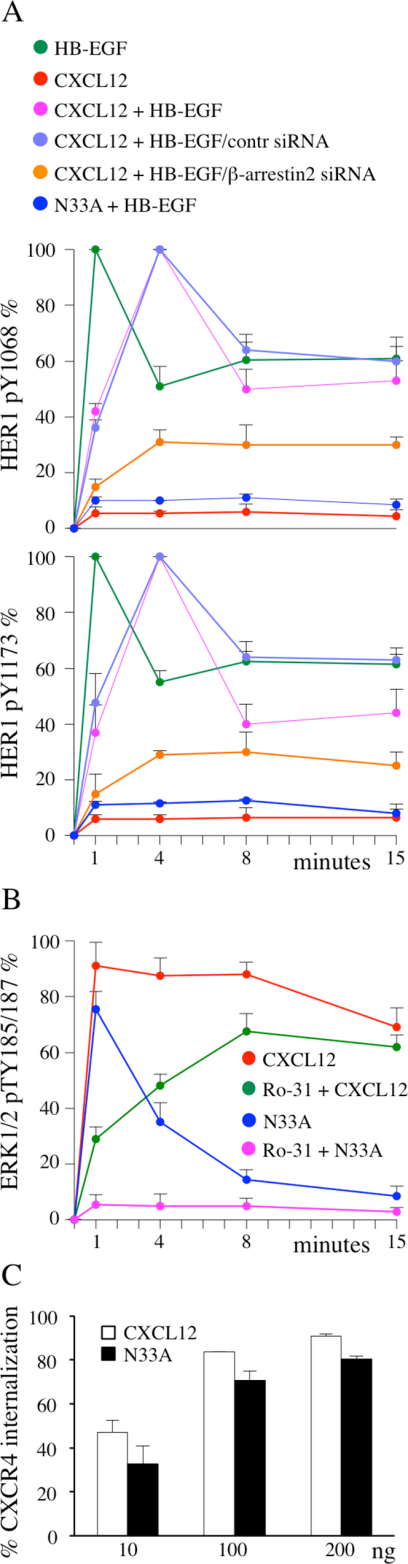
β-arrestin-dependent functions are differentially affected by CXCL12 and N33A Phosphorylations are determined by ELISA and expressed as percentages of phosphorylation at the indicated times after normalization as phosphorylated molecule/total molecule ratios. **(A)** HER1 phosphorylation at Y1068 (top) and Y1173 (bottom) in HeLa cells induced by the indicated treatments, as determined by ELISA. **(B)** ERK1/2 phosphorylation at pTY185/187 in HeLa cells induced by the indicated stimuli, as determined by ELISA. **(C)** CXCR4 internalization after 1 hour CXCL12 or N33A stimulation was evaluated by flow cytometry in Jurkat cells. Means ± SD of at least 5 experiments are depicted. p=ns.

In HER1/CXCR4 double-positive cells, HER1 transactivation/transinhibition mechanisms account for the fine tuning of epithelial growth factors signaling. In our previous work, we demonstrated that wild type CXCL12 transinhibited HB-EGF-stimulated HER1 in a G-protein dependent fashion (Figure 4A, green, red, magenta), while subsequent recruitment of β-arrestin restored HER1 phosphorylation [21]. Such an effect can be considered as an example of G protein uncoupling and canonical signaling termination. Here we observed that N33A transinhibited HB-EGF-stimulated HER1 at 1 minute, without restoring its phosphorylation at 4 minutes (Figure 4A, dark blue), indicating a clear defect in β-arrestin-dependent G protein-coupled receptor (GPCR) desensitization. Notably, if we silenced β-arrestin2 in HeLa cells before CXCL12 stimulation, some degree of HER1 transinhibition persisted over 4 minutes (Figure 4A, orange, light blue), confirming the central role that β-arrestin plays in termination of this specific G protein-triggered event.

MAPK pathway can be activated by both G protein and β-arrestin engagement [28, 29]. Upon CXCL12 stimulation we observed a sequential phosphorylation at T185/Y187 of ERK1/2 (Figure 4B, red). The protein kinase C (PKC) inhibitor Ro-31 contributed to reveal two distinct components in ERK1/2 activation: an early PKC-dependent phase, peaked at 1 minute, and a later PKC-independent, β-arrestin-induced, phase reaching the maximum at 8 minutes (Figure 4B, red, green). Upon N33A stimulation we recorded only the first, G protein dependent, spike (Figure 4B, dark blue, magenta), suggesting that β-arrestin recruitment by the complex N33A/CXCR4 did not lead to ERK1/2 phosphorylation.

Receptor endocytosis is a regulated process triggered by multi-step interactions among GPCR/β-arrestin complex, clathrin and its adaptor protein AP2 [30]. To evaluate if CXCL12 and its structural variant differed in inducing receptor internalization, we analyzed surface CXCR4 expression before and after stimulation. Although the mutant ligand resulted slightly less effective, there was no statistically significant difference between CXCL12 and N33A in mediating receptor internalization (Figure 4C).

**Figure 5:**
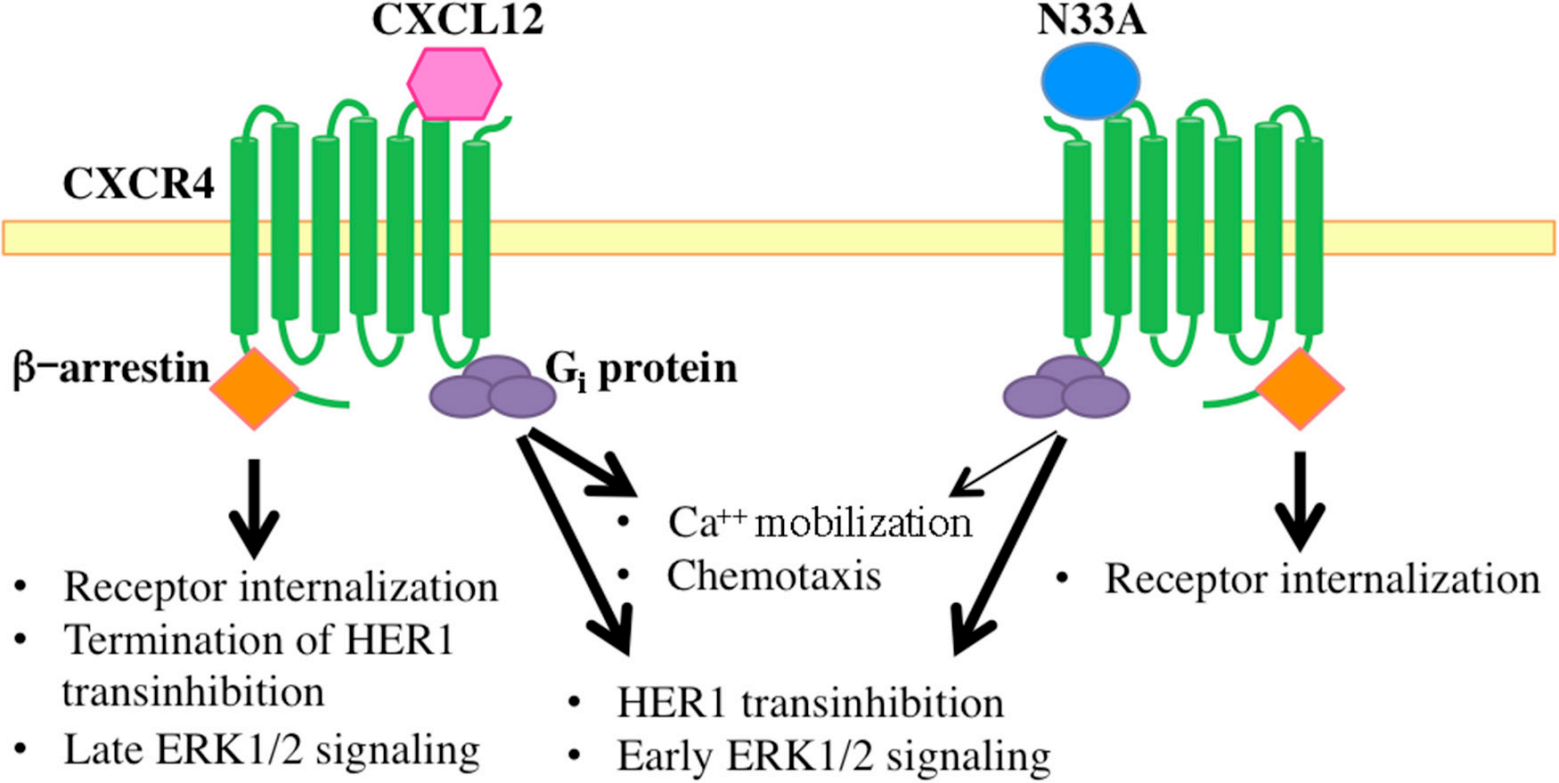
Schematic representation of intracellular signals triggered by CXCL12 and N33A Both chemokines activate heterotrimeric G_i_ proteins and related downstream signaling events, though N33A acts as a weaker CXCR4 agonist in inducing Ca^++^ mobilization and cell chemotaxis (thin arrow). N33A, which exhibits functional selectivity within the β-arrestin layer, only triggers receptor internalization without terminating HER1 transinhibition or inducing late ERK1/2 signaling.

Taken together, these findings demonstrate that the wild type ligand can indistinctly activate all the β-arrestin-dependent responses, whereas the mutant one preferentially triggers the internalization module, acting as a biased agonist within the β-arrestin level. Figure 5 gives a graphic synopsis of our findings.

## DISCUSSION

The original need of rapidly producing a large quantity of CXCL12 for research purposes led to the chemical synthesis of a structural variant in which Asn^33^ was substituted by Ala. One previous study reported that N33A displayed full biological activity through the interaction with its cognate receptor CXCR4, thus resulting as effective as the recombinant chemokine in receptor binding and peripheral blood leukocytes chemotaxis [25]. However, even a single substitution within the amino acid sequence can significantly affect protein tertiary structure and modify ligand-receptor contact points, subsequent receptor conformation and its specific pattern of activation. Our previous work demonstrated that N33A could not reverse G protein-triggered HER1 transinhibition, indirectly suggesting that it might act as a G protein biased ligand [21].

In the current study, we sought to better investigate whether CXCL12 and its mutant analogue were different in terms of G protein coupling and arrestin binding, taking into account G protein-dependent and independent cell functions as well. Although receptor binding affinity was the same, in CHO cells N33A exhibited reduced potency both in heterotrimeric G protein coupling and β-arrestin recruitment as compared to the recombinant chemokine, acting as a weaker CXCR4 ligand.

As a result of reduced potency in coupling to Gα_i_, calcium fluxes and chemotaxis, two G protein-triggered events, were significantly less increased upon N33A stimulation in Jurkat cells and, besides merely quantitative effects, the different amplitude of intracellular calcium flux induced by low concentrations of CXCL12 and N33A may elicit qualitatively different global cell responses [29]. Conversely, such a pattern of *dim* responses was not observed within β-arrestin-dependent functions. It is increasingly recognized that β-arrestins are critical molecules in regulating 7TMRs signaling and trafficking. Once recruited to the intracellular C-terminus, they mediate G protein desensitization, receptor endocytosis in clathrin-coated pits and non canonical – G protein independent – signaling, being able to compartmentalize 7TMRs response in time and space [28, 31]. Over the last ten years, Lefkowitz and coworkers have provided mounting evidence that different agonists lead to different receptor conformations, which in turn recruit specific GRKs to phosphorylate the intracellular receptor tail. Since each GRK generates a peculiar *barcode* by adding phosphate groups at distinct sites, different conformations of receptor-bound β-arrestin occur and trigger divergent downstream responses [11, 26]. Although we cannot provide proteomic-based definitive proofs about CXCR4-GRK-arrestin interaction mechanisms, our finding that N33A activates only β-arrestin-dependent receptor internalization module without inducing termination of G protein-dependent HER1 transinhibition nor triggering late ERK1/2 signaling may be explained by assuming that it could set up a distinct β-arrestin interactome clearly favoring CXCR4 endocytosis over the other pathways. While some groups have already identified G protein-biased ligands at CXCR4 [20], to our knowledge there are no previous reports about CXCR4 ligands preferentially triggering one specific β-arrestin signature. Taking into account these concepts, functional selectivity at CXCR4 progressively becomes more complex: to accurately study the effect of a receptor agonist, the evaluation of classical G protein versus β-arrestin bias principle cannot be dissociated from the investigation of the specific β-arrestin dependent modules. If we had not considered them, Gα_i_ coupling and β-arrestin binding dose-response curves would have provided too simplistic results, leading to the incorrect conclusion that N33A only exhibited reduced potency as compared to the wild type molecule. Instead, the differences highlighted within the β-arrestin layer can heavily affect the ultimate cell responses. In HeLa cells, N33A-dependent abolishment of late ERK1/2 phosphorylation spike converts a sustained ERK1/2 activation pattern into a transient one, thus potentially contributing to the regulation of the balance between cell differentiation and proliferation [32, 33]. Indeed, the notion that CXCL12 is one of the most evolutionarily conserved chemokines reflects its relevance in embryogenesis, in which differentiation and proliferation thrusts have to be carefully regulated [34]. Furthermore, persistent HER1 transinhibition due to deficient G protein desensitization switches off HB-EGF signalling in a prolonged fashion, depriving cells from survival inputs triggered by growth factors. The receptor internalization module did not significantly differ between CXCL12 and its mutant variant, suggesting that also N33A/CXCR4 complex could recruit β-arrestin in an endocytosis-permitting conformation, subsequently interacting with clathrin and its adaptor proteins to create internalization pits. Since each arrestin-dependent module is governed by site-specific CXCR4 phosphorylation at intracellular tail [10, 35], we can speculate that N33A recruits different GRKs isoforms, which in turn dictate the ultimate β-arrestin conformation. In HEK293 cells, GRK3 and GRK6 recruitment at CXCR4 promotes ERK1/2 phosphorylation, whereas GRK2 hampers MAPK signaling [10]. Conversely, Barker *et al* demonstrated that CXCR4 internalization is modulated at least in part by GRK5-mediated Hsp70 interacting protein (Hip) phosphorylation at serine-346 [32]. Similar findings have been reported by other groups in different receptor systems. For instance, at angiotensin-II receptor (AT_1A_R) internalization is mediated primarily by GRK 2/3 and ERK1/2 activation is promoted by GRK5/6 recruitment [36]; such GRKs isoforms are also responsible for β-arrestin dependent ERK signaling at V2 vasopressin receptor, while GRK2 tends to abolish prolonged MAPK pathway, negatively regulating arrestin-mediated signaling [37].

Our data show that CXCL12 and N33A differently modulate HB-EGF-induced phosphorylation of HER1, the archetypal tyrosine kinase receptor (RTK), in HeLa cells. Several lines of evidence indicate that HER1 activation is regulated at multiple layers. First, HER1 ligand biochemical structure heavily affects downstream responses by inducing different receptor dimerization patterns. As a consequence, the high-affinity ligands EGF, HB-EGF, betacellulin and transforming growth factor-α (TGFα) stabilize HER1 dimerization to elicit transient ERK1/2 phosphorylation and subsequent cell proliferation; conversely, the low-affinity ligands epiregulin and epigen induce weaker HER1 dimerization and evoke sustained, differentiation-promoting, ERK1/2 signaling [38]. Second, GPCR agonists can transactivate or transinhibit HER1 both in a paracrine/extracellular manner and along intracytoplasmic pathways [39]. Indeed, we have previously demonstrated that CXCL12 either induces HB-EGF shedding in monocytes, which in turn phosphorylates HER1 in bystander cancer cells [19], and transinhibits HER1 *via* intracellular G protein-dependent signaling in CXCR4/HER1 double-positive cells [21]. The novel finding that N33A, a biased agonist for β-arrestin-dependent internalization over the other β-arrestin-mediated pathways, persistently maintains HER1 transinhibition by blocking arrestin-dependent desensitization, adds a fascinating level of RTK regulation in which biased ligands at GPCRs might induce a biased response at RTKs. Such considerations contribute to pave the way for a deeper investigation about biased agonism in cancer field, wherein substantial translational cues still lack. In cardiovascular and neuropsychological research, biased ligands have already gained a great deal of interest and agonists with functional selectivity at beta-adrenergic receptors, angiotensin II receptors and dopamine receptors have been identified [40–42]. Interestingly, such receptor systems are expressed across many human cancers as well [43–46]. Thus, using their biased ligands to trigger one G protein or β-arrestin-specific pathway, which could in turn either transactivate or transinhibit critical growth factor receptors, may represent an exciting platform to design novel antineoplastic strategies.

In conclusion, this work provides evidence that N33A is an unbiased ligand regarding Gα_i_ coupling and β-arrestin recruitment, but is a useful tool to dissect CXCR4 signaling within the β-arrestin layer, clearly showing a bias for the internalization module over the other pathways. Moreover, our results underscore the relevance of β-arrestin-dependent mechanisms in regulating ERK1/2 phosphorylation and HER1 activation. Though still far from clinical application, the idea of modulating reciprocal GPCR/RTK crosstalk by employing GPCR biased ligands may open an interesting scenario in cancer research.

## MATERIALS AND METHODS

### Cells

Jurkat and HeLa human cell lines (acute T cell leukemia and cervical cancer respectively) were purchased from DSMZ (Braunschweig, DE). Cells were cultured in RPMI-1640 (Invitrogen, Carlsbad, CA), supplemented with 10% heat-inactivated fetal bovine serum (Invitrogen), 50 U/mL penicillin and 50 μg/mL streptomycin (complete medium, CM) and maintained at 37 °C in 5% CO_2_.

CHO cells (Chinese Hamster Ovary) were cultured by Discoverx (Fremont, CA).

### Chemokines

Recombinant human CXCL12 was from Peprotech (London, UK), synthetic analogue [N33A]CXCL12 was from Peptidesynthetic (Fareham, UK), human synthetic CXCL12 COOH-terminally conjugated with Alexa Fluor 647 (CXCL12^AF647^) was obtained from Almac Sciences (Craigavon, UK).

### Flow cytometry

All flow cytometry data variously generated were acquired and analyzed by FACSCalibur cytometer (Becton Dickinson, San Jose, CA) and FlowJo 9.3.3 software (Tree Star, Ashland, OR).

### HER1 phosphorylation

Semiconfluent, 24-hour starved HeLa cells were incubated in the presence or absence 100 ng/mL of either recombinant human CXCL12 or synthetic analogue N33A for up to 15 minutes or for 1 minute before stimulation with 25 ng/mL HB-EGF for 1, 4, 8 and 15 minutes.

Cells were also seeded on 12-well plates (1.2×10^5^) and transfected 72 hours using HiPerFect Transfection Reagent and siRNA (2 nM, β-arrestin2 siRNA no. SI02776928 and AllStars negative control siRNA-Alexa Fluor 488; Qiagen, Hilden, D) as previously described^19^. Transfection efficiency (>95%) was determined by flow cytometry. After transfection, cells were starved for 4 hours and stimulated with 25 ng/mL HB-EGF for 15 minutes. Cell pellets were obtained to perform protein extractions for total HER1, HER1 pY1068 and pY1173 ELISA.

### Protein extraction and ELISA

Cell pellets were lysed for 30 minutes in 1 mL of ice-cold cell extraction buffer (Biosource, Camarillo, CA) supplemented with a protease inhibitor cocktail (Sigma, St. Louis, MO) and 1 mM PMSF (Sigma). After centrifugation at 13,000 rpm for 10 minutes at 4°C, supernatants were stored at −80°C until used.

Total HER1, HER1 pY1068 and pY1173, and total ERK1/2 and ERK1/2 pTY185/187 were tested in protein extracts by using commercially ELISA kits (Biosource, Camarillo, CA). The percentage of phosphorylation was depicted after normalization as phosphorylated molecule/total molecule ratio.

### Chemotaxis assay

Chemotaxis assay was performed as previously reported [47]. Cells were washed twice in chemotaxis buffer (RPMI 1640, 20 mM HEPES, 0.4% BSA), resuspended at a density of 5×10^6^/mL. Aliquots of 100 μL cell suspensions were seeded in the upper chamber of a 24-well, 8-μm pore polycarbonate Transwell culture insert (Costar, Cambridge, MA) and were incubated for 90 minutes at 37°C. The lower chamber was filled with 600 μL chemotaxis buffer alone or containing 10 or 100 ng/mL CXCL12 or N33A. Cells that migrated into the lower chamber through the membrane pores were resuspended and counted in cytometry for 20 seconds at a 60 μL/min flow rate. Results were expressed as percentage of migrated cells or chemotactic index (CI = cells migrating to CXCL12 or N33A/cells migrating to medium).

### Displacement assay

Cells were incubated for 30′ at 4°C with 25 ng/mL CXCL12^AF647^. After washing varying concentrations of CXCL12 or N33A were added for 30′. Thereafter, the cells were washed and analyzed by flow cytometry. The percentage of displacement of CXCL12^AF647^ by CXCL12 or N33A was calculated according to the formula

(1 - (MFI - MFI_NC_)/(MFI_AF647_ - MFI_NC_) x 100

where MFI is the mean fluorescence intensity of the cells incubated with different concentrations of CXCL12 or N33A after staining with CXCL12^AF647^, MFI_NC_ is the autofluorescence of unlabeled cells and MFI_AF647_ is the mean fluorescence intensity of the cells incubated with CXCL12^AF647^ alone [48].

### Calcium flux

To evaluate Ca^2+^ influx, cells were resuspended in HBSS/Ca^2+^ and loaded with 2 μM Fluo-4 AM (Invitrogen, Paisley, UK) for 45 minutes at 37°C. Then they were washed and analyzed by flow cytometry. After 60 seconds baseline was collected, 10 or 100 ng/mL CXCL12 or N33A were added. Time-dependent cell fluorescence was recorded at a detection wave length of 516 nm. Calcium flux response was visualized using the kinetic analysis unit in FlowJo.

### CXCR4 internalization

Jurkat cells were incubated at 2×10^6^ for 1 h at 37°C with or without 10, 100 and 200 ng/mL CXCL12 or N33A. Cells were then washed and stained for 1 h at 4°C with anti-human CXCR4-PE mAb (Becton Dickinson). The percentage of receptor internalization was expressed as the difference between basal CXCR4 MFI and CXCR4 MFI upon stimulation.

### Coupling to Gα_i_

cAMP Hunter^TM^ CHO-K1 CXCR4 G_i_ cells were seeded in 384-well microplates and incubated with CXCL12 or N33A in the presence of EC80 (15 μM) forskolin at 37°C in HBSS+5mM HEPES. cAMP concentration was determined utilizing HitHunter^®^ cAMP assay (Discoverx, Fremont, CA). Chemilunescence was measures after 3 hours at room temperature with a PerkinElmer EnvisionTM instrument (Waltham, MA). Chemiluminescence produced by each experimental point was subtracted from forskolin treated sample and expressed as percentage of the maximal inhibition produced by CXCL12.

### Arrestin binding

The PathHunter^®^ β-Arrestin assay (Discoverx) was performed to evaluate β-arrestin recruitment triggered by CXCL12 or N33A. The assay is based on complementation of β-galactosidase (β-Gal) function relying on reassembling of two complementary and inactive parts of the enzyme split and expressed as fusion proteins to CXCR4 and β-arrestin in CHO cells. β-arrestin recruitment restoring β-Gal activity was measured using chemiluminescent PathHunter^®^ Detection Reagents and PerkinElmer EnvisionTM instrument. Basal chemiluminescence produced by vehicle control was subtracted and data were expressed as percentage of CXCL12-induced maximal activity.

### Quantifying bias

All Gα_i_ coupling and β-arrestin assays data were analyzed using GraphPad Prism Software (La Jolla, CA). Curves were fitted using least squares nonlinear regressions, assuming a sigmoidal fit for dose-response curves. Bias factor of N33A for β-arrestin recruitment versus Gα_i_ engagement was calculated by the method previously published by Kenakin [27], using the *operational model* of Black and Leff [49].

### Statistics

Student's t-test for means and Kruskall-Wallis analysis of variance by rank were considered significant for p values <0.05.

## Abbreviations

N33A: [N33A]CXCL12
7TMR: seven transmembrane receptor
ACKR3: atypical chemokine receptor 3
ICL3: intracellular loop 3
MAPK: mitogen-activated protein kinase
PLC: phospholipase C
GRK: G protein-coupled receptor kinase
ERK1/2: extracellular-signal regulated kinases 1/2
PDE4: phosphodiesterase 4
HER1: epithelial growth factor receptor 1
HB-EGF: heparin-binding EGF-like growth factor
cAMP: 3′, 5′-cyclic adenosine monophosphate
CHO: chinese hamster ovary
GTP: guanosine triphosphate
GDP: guanosine diphosphate
GPCR: G protein-coupled receptor
Asn: asparagine
Ala: alanine
Hip: Hsp70 interacting protein
AT_1A_R: angiotensin-II receptor
RTK: receptor tyrosine kinase
EGF: epithelial growth factor
TGFα: transforming growth factor α
